# Inverse association of NSAID use and ovarian cancer in relation to oral contraceptive use and parity

**DOI:** 10.1038/sj.bjc.6604392

**Published:** 2008-05-27

**Authors:** K J Wernli, P A Newcomb, J M Hampton, A Trentham-Dietz, K M Egan

**Affiliations:** 1 Cancer Prevention Program, Fred Hutchinson Cancer Research Center, 1100 Fairview Avenue N, M4-B402, Seattle, WA 98109, USA; 2 Department of Cancer Control, University of Wisconsin Paul P Carbone Comprehensive Cancer Center, Madison, WI 53726, USA; 3 Department of Risk Assessment, Detection, and Intervention, H Lee Moffitt Cancer Center and Research Institute, Tampa, FL 33612, USA

**Keywords:** ovarian cancer, non-steroidal anti-inflammatory drugs, parity, oral contraceptives

## Abstract

We examined the association between non-steroidal anti-inflammatory drug (NSAID) use and ovarian cancer by potential effect modifiers, parity and oral contraceptive use, in a population-based case–control study conducted in Wisconsin and Massachusetts. Women reported prior use of NSAIDs and information on risk factors in a telephone interview. A total of 487 invasive ovarian cancer cases and 2653 control women aged 20–74 years were included in the analysis. After adjustment for age, state of residence and other covariates, ever use of NSAIDs was inversely associated with ovarian cancer in never users of oral contraceptives (odds ratio (OR)=0.58, 95% confidence interval (CI) 0.42–0.80) but not for ever users (OR=0.98, 95% CI 0.71–1.35) (*P*-interaction=0.03). A reduced risk with NSAID use was also noted in nulliparous women (OR=0.47, 95% CI 0.27–0.82) but not among parous women (OR=0.81, 95% CI 0.64–1.04) (*P*-interaction=0.05). These results suggest that use of NSAIDs were beneficial to women at greatest risk for ovarian cancer.

Over the last 10 years, a new hypothesis has emerged, suggesting that the presence of chronic inflammation might play a causal role in the development of ovarian cancer (Ness and Cottreau, 1999). Inflammation might be due to the process of ovulation itself or the presence of chronic inflammatory agents (Fleming *et al*, 2006). Non-steroidal anti-inflammatory drugs (NSAIDs) have been linked to a lower risk of several cancers (Bosetti *et al*, 2006), and NSAIDs could in theory have a similar protective influence in ovarian cancer by mitigating the deleterious effects of inflammation on ovarian epithelium. Results of several observational studies and one randomised trial (Cook *et al*, 2005) of NSAIDs and ovarian cancer risk are inconsistent. Five small studies detected a modestly decreased risk, but lacked statistical power (Cramer *et al*, 1998; Rosenberg *et al*, 2000; Akhmedkhanov *et al*, 2001; Lacey *et al*, 2004; Schildkraut *et al*, 2006). Five studies (Tavani *et al*, 2000; Moysich *et al*, 2001; Fairfield *et al*, 2002; Meier *et al*, 2002; Friis *et al*, 2003) plus a meta-analysis (Bonovas *et al*, 2005) have demonstrated no association with aspirin or other non-aspirin NSAID use. One study suggested an increased risk (Sorensen *et al*, 2003). Lack of consistent findings in prior studies of NSAID use could reflect heterogeneity across study populations on factors such as parity and oral contraceptive use, which may reduce inflammation by suppressing ovulation. We explored the relationship of NSAID use with ovarian cancer risk, and whether NSAID associations depended on potential interactions with oral contraceptive use and parity in a US population-based case–control study.

## MATERIALS AND METHODS

The study has been described in detail in previous reports (Peterson *et al*, 2006). Briefly, women aged 20–74 years diagnosed with incident invasive epithelial ovarian cancer (ICDO-C56) between 1998 and 2001 from Wisconsin and Massachusetts were identified through state-wide cancer registries. Controls were randomly selected in each state using two sampling frames: (1) for women <65 years, a list of licensed drivers; or (2) for women ⩾65 years, rosters of Medicare beneficiaries compiled by the Centers for Medicare and Medicaid Services. All eligible controls had a publicly available telephone number. Controls were frequency-matched within 5-year stratum to the age distribution of ovarian and breast cancer cases enrolled in a concurrent study (Sprague *et al*, 2007). All study participants provided informed consent to participate. Response rates were 67% for cases and 82% for controls.

All eligible women were mailed an introductory study letter before contact by study personnel. A trained interviewer conducted a 45-min telephone survey, which elicited information on use of NSAIDs, including the frequency, start and stop dates, duration of use, and type. Only episodes of use before a reference date were included; the reference date was the date of diagnosis for cases and the date 1 year before interview for controls. Non-steroidal anti-inflammatory drugs included both aspirin types and non-aspirin NSAID types (e.g., ibuprofen). ‘Ever use’ was defined as the use of any NSAID for at least twice per week for 6 months or more. A woman was defined as a ‘current user’ of NSAIDs if she reported use within 12 months of the reference date and lasting at least 6 months in duration. ‘Former use’ was defined as use for at least 6 months duration, but before the 12 months preceding the reference date. ‘Never use’ was defined as use of NSAIDs for less than 6 months or less than twice per week. Information on other risk factors for ovarian cancer, including oral contraceptive use and parity, were also elicited during the interview. Cases (*n*=18) and controls (*n*=562) were excluded if they had missing data on NSAID use. The final sample included 487 cases and 2653 controls.

Multivariable logistic regression analysis was used to calculate odds ratios (OR) and 95% confidence intervals (CIs) from Stata 9 (Stata Corp, College Station, TX, USA) (Breslow and Day, 1980). The main model included terms for age, state of residence, study year, menopausal status, body mass index, parity, oral contraceptive use, history of tubal ligation, hysterectomy and family history of ovarian cancer. Tests for trend with two-sided *P*-values were evaluated by entering the categorical terms as an ordinal variable in the model. To test for interaction of NSAIDs with oral contraceptive use and parity, a cross-product term was entered into the model, and the likelihood ratio test was used to test the difference between the full model and reduced model.

## RESULTS

Case women were similar to control women with respect to age, education, age at first birth and smoking status (Table 1). Cases were more likely than control women to have not used oral contraceptives, be nulliparous or postmenopausal, or have had a tubal ligation or a family history of ovarian cancer.

Overall, we observed a reduction in risk among women who had ever used any type of NSAID compared to non-users (OR=0.74, 95% CI 0.59–0.92) (Table 2). When considered according to oral contraceptive use, ever use of NSAIDs was inversely associated with ovarian cancer in never users (OR=0.58, 95% CI=0.42–0.80), but there was no association among ever users (OR=0.98, 95% CI=0.71–1.35) of these medications (*P*-interaction=0.03) (Table 2). The reduction in risk was seen for both current and former users among never oral contraceptive users. A reduced risk with NSAID use was limited to nulliparous women (OR=0.47, 95% CI=0.27–0.82), whereas there was little evidence of association among parous women (OR=0.81, 95% CI=0.64–1.04) (*P*-interaction=0.05). There was a significant decreasing trend in risk with increasing years of NSAID use among nulliparous women alone (*P*-trend=0.01).

## DISCUSSION

Results from this study indicate an overall 30% reduction of ovarian cancer associated with ever and current use of NSAIDs. Further, NSAID users who are nulliparous or have not used oral contraceptives had the greatest reductions in risk. Both pregnancy and use of oral contraceptives are associated with decreased ovarian cancer risk through their inhibitory effects on ovulation, due to incessant ovulation (Fathalla, 1971). At the site of ovum release, ovulation is associated with leukocyte invasion, release of nitric oxide and inflammatory cytokines, vasodilation, DNA repair, and tissue remodelling (Fleming *et al*, 2006). The use of NSAIDs could potentially mitigate the inflammatory effect of ovulation, possibly at the site of ovarian inclusion cysts.

There are some limitations that should be considered in the present analysis. We relied on the subject's ability to recall the use of NSAIDs, which could have introduced misclassification and potential bias towards the null. Although there are reports suggesting that age and education might influence recall, it has been shown that more frequent use of NSAIDs is more reliably recalled than less frequent use (Lewis *et al*, 2006; West *et al*, 1995). Therefore, to improve the sensitivity, we attempted to identify true and consistent users by limiting ever use of NSAIDs to at least two tablets per week for 6 months or more.

These results suggest that NSAIDs may confer greater protection from ovarian cancer in women with higher background levels of inflammation such as those from incessant ovulation.

## Figures and Tables

**Table 1:** Demographic characteristics of ovarian cancer cases and population-based controls

	**Cases (*n*=487)**	**Controls (*n*=2653)**
**Demographic characteristics**	***N* (%)**	***N* (%)**
Mean age (s.d.), years	55 (11.6)	54 (10.1)
High school degree	196 (42.8)	1070 (42.8)
Overweight or obese	272 (55.9)	1374 (51.8)
Oral contraceptive use	220 (45.5)	1411 (53.7)
Mean age (s.d.) at first birth, years	23.9 (4.5)	23.8 (4.5)
Nulliparous	105 (21.6)	311 (11.7)
Postmenopausal	309 (63.4)	1425 (53.7)
Ever smoker	253 (52.0)	1402 (52.8)
Tubal ligation	87 (17.9)	653 (24.6)
Hysterectomy	107 (22.0)	380 (14.3)
Family history of ovarian cancer	24 (4.9)	70 (2.6)

**Table 2:** Risk of ovarian cancer associated with NSAID use overall and stratified by oral contraceptive use and parity

	**Cases**	**Controls**		**Cases**	**Controls**		
**NSAID use**	***N* (%)**	***N* (%)**	**OR (95% CI)^a^**	***N* (%)**	***N* (%)**	**OR (95% CI)a,b**	***P*-interaction**
		*Overall*					
Never use	336 (70.7)	1711 (65.7)	1.00 (Referent)				
Ever use	139 (29.3)	893 (34.3)	0.74 (0.59–0.92)				
Former	45 (9.6)	220 (8.6)	0.96 (0.67–1.37)				
Current	90 (19.1)	637 (24.8)	0.68 (0.52–0.88)				
							
Aspirin only	64 (16.0)	396 (18.8)	0.73 (0.54–1.00)				
Ibuprofen only	59 (14.9)	424 (19.9)	0.68 (0.50–0.93)				
							
Duration (years)							
<1	29 (6.1)	189 (7.3)	0.74 (0.49–1.14)				
1–4	50 (10.5)	378 (14.5)	0.64 (0.46–0.88)				
5–9	30 (6.3)	150 (5.8)	0.94 (0.61–1.44)				
⩾10	30 (6.3)	176 (6.8)	0.78 (0.50–1.19)				
*P-trend*			*0.03*				
							
	*Never used oral contraceptives*			*Ever used oral contraceptives*			
	(*n*=276)	(*n*=1483)		(*n*=222)	(*n*=1669)		
Never use	189 (74.1)	758 (63.7)	1.0 (Referent)	144 (66.4)	935 (67.4)	1.0 (Referent)	
Ever use	66 (25.9)	432 (36.3)	0.58 (0.42–0.80)	73 (33.6)	453 (32.6)	0.98 (0.71–1.35)	0.03
Former	18 (7.1)	94 (8.0)	0.69 (0.40–1.20)	27 (12.6)	125 (9.1)	1.32 (0.82–2.12)	0.1
Current	46 (18.2)	321 (27.4)	0.56 (0.39–0.80)	44 (20.5)	309 (22.6)	0.85 (0.58–1.26)	
							
Aspirin only	38 (15.0)	218 (18.4)	0.63 (0.42–0.93)	26 (12.0)	175 (12.7)	0.97 (0.60–1.57)	0.04
Ibuprofen only	20 (7.9)	180 (15.2)	0.45 (0.27–0.75)	39 (18.1)	239 (17.3)	0.95 (0.63–1.43)	
							
Duration (years)							
<1	15 (5.9)	89 (7.5)	0.67 (0.37–1.21)	14 (6.5)	98 (7.1)	0.89 (0.48–1.65)	0.04
1–4	20 (7.8)	188 (15.8)	0.42 (0.26–0.69)	30 (13.8)	187 (13.5)	0.96 (0.61–1.49)	
5–9	14 (5.5)	76 (6.4)	0.62 (0.33–1.14)	16 (7.4)	72 (5.2)	1.56 (0.86–2.85)	
⩾10	17 (6.7)	79 (6.6)	0.82 (0.46–1.45)	13 (6.0)	96 (6.9)	0.71 (0.36–1.39)	
*P-trend*			*0.008*			*0.85*	
							
	*Nulliparous*			*Parous*			
	(*n*=110)	(*n*=382)		(*n*=392)	(*n*=2811)		
Never use	79 (78.2)	197 (64.4)	1.0 (Referent)	257 (68.7)	1514 (66.0)	1.0 (Referent)	
Ever use	22 (21.8)	109 (35.6)	0.47 (0.27–0.82)	117 (31.3)	781 (34.0)	0.81 (0.64–1.04)	0.05
Former	6 (6.0)	32 (10.6)	0.42 (0.16–1.08)	39 (10.5)	188 (8.3)	1.15 (0.78–1.68)	0.1
Current	15 (15.0)	72 (23.9)	0.48 (0.25–0.94)	75 (20.2)	562 (24.8)	0.72 (0.54–0.96)	
							
Aspirin only	8 (7.9)	40 (13.2)	0.48 (0.20–1.11)	56 (15.1)	355 (15.5)	0.78 (0.57–1.09)	0.3
Ibuprofen only	12 (11.9)	60 (19.8)	0.45 (0.22–0.92)	47 (12.7)	362 (15.8)	0.77 (0.55–1.08)	
							
Duration (years)							
<1	7 (6.9)	20 (6.5)	0.80 (0.32–2.03)	22 (5.9)	167 (7.3)	0.75 (0.46–1.20)	0.03
1–4	6 (5.9)	51 (16.7)	0.30 (0.12–0.74)	44 (11.8)	326 (14.2)	0.72 (0.51–1.03)	
5–9	4 (4.0)	17 (5.6)	0.54 (0.17–1.74)	26 (7.0)	133 (5.8)	1.06 (0.67–1.67)	
⩾10	5 (5.0)	21 (6.9)	0.47 (0.15–1.48)	25 (6.7)	155 (6.8)	0.88 (0.55–1.39)	
*P-trend*			*0.01*			*0.25*	

a Adjusted for age, year of interview, state of residence, tubal ligation, family history of ovarian cancer, hysterectomy and menopausal status.

b Each analysis is adjusted for the other two variables in the table.
